# Modern and traditional approaches combined into an effective gray-box mathematical model of full-blood acid-base

**DOI:** 10.1186/s12976-018-0086-9

**Published:** 2018-09-10

**Authors:** Filip Ježek, Jiří Kofránek

**Affiliations:** 1 Department of Cybernetics, Faculty of Electrical Engineering, Czech Technical University in Prague, Prague, Czech Republic; 2 Institute of Pathological Physiology, First Faculty of Medicine, Charles University, U nemocnice 5, 128 00 Prague 2, Czech Republic

**Keywords:** Acid-base modeling, Physicochemical acid-base, Behavioral acid-base, Siggaard-Andersen, Modelica, Physiology, Physiolibrary

## Abstract

**Background:**

The acidity of human body fluids, expressed by the pH, is physiologically regulated in a narrow range, which is required for the proper function of cellular metabolism. Acid-base disorders are common especially in intensive care, and the acid-base status is one of the vital clinical signs for the patient management. Because acid-base balance is connected to many bodily processes and regulations, complex mathematical models are needed to get insight into the mixed disorders and to act accordingly. The goal of this study is to develop a full-blood acid-base model, designed to be further integrated into more complex human physiology models.

**Results:**

We have developed computationally simple and robust full-blood model, yet thorough enough to cover most of the common pathologies. Thanks to its simplicity and usage of Modelica language, it is suitable to be embedded within more elaborate systems. We achieved the simplification by a combination of behavioral Siggaard-Andersen’s traditional approach for erythrocyte modeling and the mechanistic Stewart’s physicochemical approach for plasma modeling. The resulting model is capable of providing variations in arterial pCO2, base excess, strong ion difference, hematocrit, plasma protein, phosphates and hemodilution/hemoconcentration, but insensitive to DPG and CO concentrations.

**Conclusions:**

This study presents a straightforward unification of Siggaard-Andersen’s and Stewart’s acid-base models. The resulting full-blood acid-base model is designed to be a core part of a complex dynamic whole-body acid-base and gas transfer model.

**Electronic supplementary material:**

The online version of this article (10.1186/s12976-018-0086-9) contains supplementary material, which is available to authorized users.

## Background

Acid-base disturbances are associated with a number of fluid, electrolyte, metabolic and respiratory disorders. Understanding the pathogenesis and the underlying pathophysiological processes is crucial for proper diagnostics and treatment, especially in acute medicine, anaesthesiology and during artificial respiration. Complex mathematical models help to uncover the pathogenesis of complex bodily disorders.

Two major approaches for mathematical modeling of the acid-base status of blood are widely employed. The traditional model by Siggaard-Andersen [1] (SA) is a behavioral model of full blood acid-base (i.e. including erythrocytes), but it is originally defined for standard albumin and phosphates only (though added later [2]) and, on its own, the model is unable to assess hemodilution and hemoconcentration (e.g. during fluid replacement), and the individual levels of ions are not considered. The second model, Stewart’s physicochemical [3] model, or the so-called modern approach, is a structural model of plasma only, but it is essential for assessing hemodilution, ion and protein imbalances, which are common in critically ill patients. However neither of these is satisfactory as a complete model.

The Stewart’s physicochemical approach has been further extended to overcome some of its disadvantages. Rees and Andreasen shown the extension to full blood and enhanced it by circulation, blood gases, interstitium and cellular compartments [4]. Wooten later presented an extension to the extracellular compartment [5] and most recently, Wolf [6] proposed a profound steady-state physicochemical model of erythrocyte-plasma-interstitium-cell compartments, including detailed ion and water balance. It has been built with the purpose to mechanistically describe complex physicochemical processes, which is, however, computationally demanding and hard to solve due to a large system of non-linear equations. This prevents us from integrating the detailed models into more complex models, or from usage for patient-specific identification.

Described approaches [1–6] are, however, designed for steady-state situations. To extend the whole-body acid-base assessment by bodily regulatory loops and to show pathogenesis of developing disorders in time, one needs to construct even larger integrative model, including important bodily compartments (interstitial fluid, cells) and regulations (kidney, liver, respiratory regulation) interconnected by a circulation of full-blood, the core component of our approach [7]. From the authors’ experience, these subcomponents however need to be based on computationally robust submodels, which are yet precise enough to describe the physiology.

The aim of this study is to develop a detailed, yet computationally effective, full blood model by combining the two predominant approaches to blood acid-base balance. The resulting combined model will become an essential part of a future complex dynamic whole-body acid-base and blood gas transfer model following the border flux theory [7] to assess dynamic bodily compensations.

## Methods

**Fig. 1:**
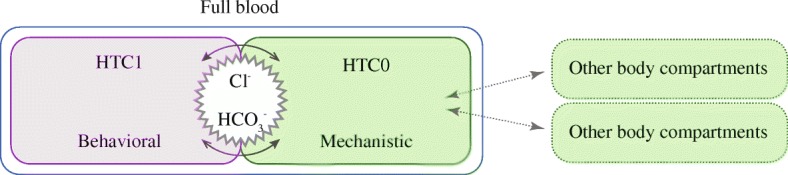
Diagram of described full blood model, connected to other body compartments. The HCT1 compartment contains erythrocytes (blood with hematocrit reaching 1) and is modeled behaviorally, whereas the plasma (HCT0) is described mechanistically. Other body compartments are connected directly to plasma

If the detailed processes in erythrocyte are not the objective, an empirical description of their contribution is often satisfactory, which is substantially lowering the model complexity. Therefore, instead of modeling erythrocyte mechanistically, we propose to substitute it with behavioral description, whereas the plasma should follow the physicochemical mechanistic design to include ion transfer to adjacent body compartments (Fig. 1). This approach would simplify complex physicochemical computations of membrane balance.

The Siggaard-Andersen’s (SA) approach (also known as the traditional approach), built around SA nomogram [8], uses measured values of pH and arterial pCO_2_ to compute *buffer base* (BB), a concentration of buffer anions and cations, which can take a buffering action. A difference between BB and *normal buffer base* (NBB) is called *base excess* (BE), which expresses how many milimoles of strong acid must be added to 1 l of blood to regain normal pH (at normal pCO_2_ = 40 mmHg). More recently, the term BE has been substituted by the Concentration of Titratable Hydrogen Ion (ctH^+^), which however equals to the negative of BE [9]. The BE proved to be a handy indicator for the clinicians to quickly assess the level of metabolic acid-base disturbance. In the following years, the importance of the printed acid-base nomogram declined in favor of its numerical formalization, e.g. the Van Slyke eq. [9], so that we can form a function for Siggaard-Andersen’s pH as:

1 $$ {\mathrm{pH}}_{\mathrm{SA}}={\mathrm{f}}_{\mathrm{SA}}\left(\mathrm{BE},{\mathrm{pCO}}_2,\mathrm{Hb}\right) $$

The physicochemical, (also referred to as a modern or Stewart’s approach), does not rely on measured nomogram. Instead, a so-called strong ion difference (SID) is calculated from the difference of strong (i.e. mostly or fully dissociated at physiologic ranges) cations and anions concentrations (which equals charge for fully-dissociated substances) in plasma. Because of the electroneutrality principle, the SID must equal the sum of charges of HCO^−^ _3_, phosphates and albumin. SID can be calculated from the difference of ion concentrations, i.e. [Na^+^] + [K^+^] + 2[Mg^2+^] + 2[Ca^2+^] - Cl^−^ - lactate^−^ (but sometimes simplified to [Na^+^] + [K^+^] + 2[Mg^2+^] + 2[Ca^2+^] - Cl^−^ for clinical use) and is then known as an *apparent SID* (SIDa) for omitting the role of weak acids. Or on the contrary, the SID is calculated from approximation of HCO^−^ _3_, phosphates and albumin charges, called then the *effective SID* (SIDe) [10]. The difference between SIDa and SIDe comprises the unmeasured anions (sulfate, keto-acids, citrate, pyruvate, acetate and gluconate). For computer modeling, we consider that SIDe = SID. The pH is then believed to be specified physicochemically by a function:

2 $$ {\mathrm{pH}}_{\mathrm{PC}}={\mathrm{f}}_{\mathrm{PC}}\left(\mathrm{SID},{\mathrm{pCO}}_2,\mathrm{Alb},\mathrm{Pi}\right) $$

where the Alb and Pi are the plasma concentrations of the albumin and phosphates, respectively. The main idea of our approach is to divide the blood into two theoretical limit compartments. The first compartment is containing only erythrocytes (HCT1, i.e. with hematocrit 1) and the other, only plasma (HCT0). Some ions may move between the two compartments.

The HCT1 compartment is described by the behavioral Siggaard-Andersen model [1], extrapolated to full hematocrit (of fully oxygenated blood). This is satisfactory, as we are not interested in the inner state of the erythrocyte. On the contrary, the HCT0 compartment is governed by the mechanistic physicochemical model, following the Stewart’s approach. When the two compartments are mixed together into full-blood, their pH must be equal:

3 $$ {\mathrm{pH}}_{\mathrm{HCT}1}={\mathrm{pH}}_{\mathrm{HCT}0}={\mathrm{pH}}_{\mathrm{BLOOD}} $$

where pH_HCT1_ = pH_SA_ and pH_HCT0_ = pH_PC_. Note that pH_HCT1_ does not represent the pH inside an erythrocyte, as modeled by Raftos et al., Rees et al. or Wolf and DeLand [11–13]. In contrast, it is a limit of the pH outside erythrocytes, as the hematocrit of this theoretical compartment (the ratio of erythrocytes) approaches 1. For computation of pH_HCT1_, we employ our formalization of Siggaard-Andersen’s nomogram, using a set of three sixth-order and a fourth order polynomials, whereas pH_HCT0_ (effectively plasma only) is given by Fencl’s simple plasma description [14]. However, the particular models used can be replaced by more complex descriptions, with some trade-off between level of detail and numerical complexity. Please refer to the online supplement for the details regarding the calculation of pH_HCT1_ by own formalization of Siggaard-Andersen’s nomogram (eqs. 1–11 in the Additional file 1) and on calculation of pH_HCT0_ from the physicochemical model (eqs. 12–16 in the Additional file 1).

Interconnection of these two compartments is made possible by the definition of BE_PC_ (base excess calculated for **p**hysico-**c**hemical domain). Similar to the idea of SIDEx (or *SID excess*) [15] and analogically to the definition of normal buffer base (NBB) [8], let the *normal SID* (NSID) be the SID under standard conditions, i.e. pH 7.4 and pCO_2_ 5.32 kPa (40 mmHg) at actual levels of total phosphates (Pi) and albumin (Alb) concentrations, so that we can assemble a function f_NSID_:

4 $$ \mathrm{NSID}={\mathrm{f}}_{\mathrm{NSID}}\left(\mathrm{Alb},\mathrm{Pi}\right),\mathrm{at}\ \mathrm{pH}=7.4,{\mathrm{pCO}}_2=5.32\ \mathrm{kPa} $$

For computation of f_NSID_ we employ the physicochemical model with Alb, Pi, pH and pCO_2_ as inputs and SID as an output. For derivation of such function, please refer to the eqs. 16–18 of the online supplement. Then, in addition to Wooten’s ΔBE = ΔSID [16] and again, analogically to definition of Siggaard-Andersen’s BE_SA_ = BB - NBB [17], we define the BE_PC_ as:

5 $$ {\mathrm{BE}}_{\mathrm{PC}}=\mathrm{SID}\hbox{-} \mathrm{NSID}={\mathrm{BE}}_{\mathrm{HCT}0} $$

BE_HCT0_ (Base excess of the zero-hematocrit compartment) is formed by the physicochemical model of plasma, so in this case it equals BE_PC_.

Now, when we mix the virtual limit HCT0 and HCT1 compartments, the mixture must establish a new BE (that is the BE of the whole blood, further referred to as BE unless stated otherwise) by a well-known Cl^−^ for HCO_3_^−^ passive exchange between the HCT0 and HCT1 compartments (that is shift of HCO_3_^−^ ions in the Siggaard-Andersen approach, or Cl^−^ in Stewart’s terms, for further considerations see the Discussion section). Based on this assumption, we have developed the following equations to quantify the total charge of transferred ions Z_TI_ (meq/l) ion transport between compartments:

6 $$ {\mathrm{BE}}_{\mathrm{HCT}0}=\mathrm{BE}\hbox{-} {\mathrm{Z}}_{\mathrm{TI}}\times \left(1\hbox{-} \mathrm{Hct}\right) $$

7 $$ {\mathrm{BE}}_{\mathrm{HCT}1}=\mathrm{BE}+{\mathrm{Z}}_{\mathrm{TI}}\times \mathrm{Hct} $$

where the BE_HCT1_ is the BE of the HCT1 compartment (all erythrocytes) and hematocrit (Hct, unitless) represents the size of the HCT1 compartment and scales the ion transfer accordingly.

The set of eqs. (1–7) leads to an iterative numerical solution, but we take advantage of the Modelica language and let the Modelica tool (Dymola 2016, Dassault Systémes) find the solution of the BE – pH relationship. Due to the possibility of Modelica to interchange the input with output, we can form a function for BE:

8 $$ \mathrm{BE}={\mathrm{f}}_{\mathrm{CombinedModelBE}}\left(\mathrm{pH},{\mathrm{pCO}}_2,\mathrm{Alb},\mathrm{Pi}\right) $$

where pH is the independent variable (previously known or varied) or a function for pH:

9 $$ \mathrm{pH}={\mathrm{f}}_{\mathrm{CombinedModelpH}}\left(\mathrm{BE},{\mathrm{pCO}}_2,\mathrm{Alb},\mathrm{Pi}\right) $$

where the BE is the independent property (known or varied). The illustrative simplest complete Modelica source code for this case is listed in Appendix.

A Modelica tool (tested in Dymola 2016 by Dassault Systémes and OpenModelica 1.11 by OpenModelica Consortium) automatically solves the set of coupled equations, employs necessary numerical methods and finds the steady-state solution.

The model is then validated by a visual comparison with the contemporary models in use.

We executed a steady-state sensitivity analysis of the buffer capacity for albumin and phosphate concentration levels on the combined model in comparison with contemporary models. The initial plasma concentrations were varied from 50 to 200% of the nominal value (4.4 g/dl for albumin and 1.15 g/dl for phosphate). The BE was held constant during the changes; therefore, according to eq. (5), the plasma SID was also varied.

Dilution by saline solution is simulated by the multiplication of SID by a given dilution factor. We then compute BE as

10 $$ \mathrm{BE}=\mathrm{Nominal}\_\mathrm{SID}\times \mathrm{DilutionFactor}-\mathrm{NSID} $$

where the NSID is, according to the above definition, a normal SID for the actual (i.e. here diluted) Alb and Pi, and the *Nominal_SID* is the SID preceding dilution. The hemoglobin is also diluted by the same factor.

For the lack of established metric to compare the computational complexity and solvability of equation-based models, the former is demonstrated by a sum of non-trivial equations and the latter by the initialization time (through the initial value of *CPU time* variable, provided by the Modelica tool). We show the values of our Combined model compared to our Modelica implementation of the Wolf model, as a representative of a complete physicochemical approach. The models were compared in Dymola 2016, on a reference computer with Windows 10 64b and i7-3667 U processor. To correctly count small time spans, each model was run 1000 times in parallel and then the CPU time was divided by the same factor.

The model source code implemented in the Modelica language, including our implementation of the Wolf’s model and source codes for the figures, is accessible at [18].

## Results

The main result of the present study is the combination of the Siggaard-Andersen and Stewart’s physicochemical models into a single model, so that we can perform calculations for dilution, albumin, phosphate and the buffer capacity of erythrocytes within a joint computationally effective combined model. The secondary result is the definition of NSID, an indicator showing the relation of BE and SID, each a flagship of its own approach. Thanks to NSID, we can quantify shifts in BE due to the dilution and/or changes in albumin levels or differences in SID due to varying pCO_2_ in full blood.

**Fig. 2:**
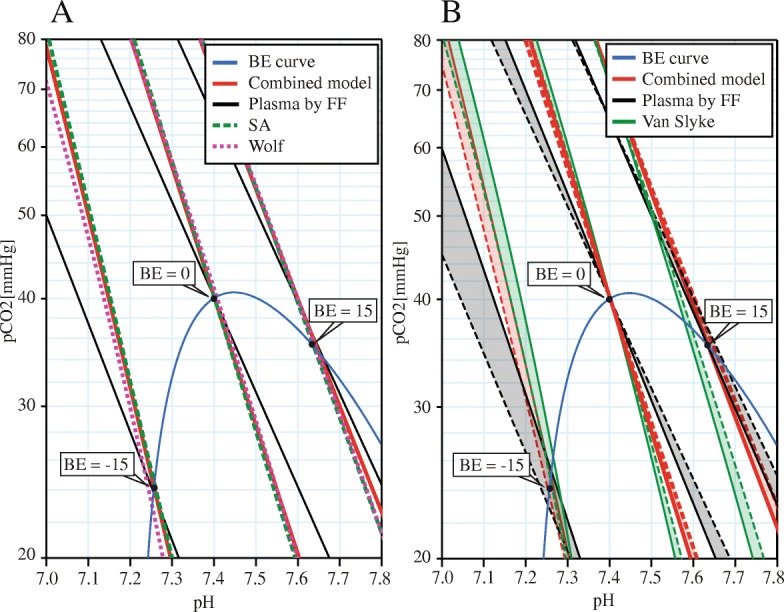
Results of Combined model in the pH-pCO_2_ diagram. **a** Comparison of our Combined model with the formalised SA nomogram reflecting full blood, with the Figge-Fencl (FF) model of plasma and with the recent full blood model by Wolf [6] reduced to the erythrocyte-plasma compartments. **b** Sensitivity analysis for albumin of our combined model, the updated Van Slyke Eq. [18] and the Figge-Fencl (FF) model of plasma

We validate the combined model by comparison with the Siggaard-Andersen’s nomogram [8], later Siggaard-Andersen’s updated albumin-sensitive Van Slyke formalization [19], Figge-Fencl’s physicochemical model (FF) [14], updated to version 3.0 [20]) and the recent full blood model by Wolf [6] (reduced to the plasma-erythrocyte compartments). A graphic comparison (Fig. 2a) shows that the Combined model fits the Siggaard-Andersen’s model, as well as the more recent model of Wolf, whereas the Figge-Fencl physicochemical model of plasma alone substantially differs from full blood models.

The results of sensitivity analysis at various plasma albumin concentrations at constant BE are outlined in Fig. 2b (compared with the Figge-Fencl model and the later albumin-sensitive Van-Slyke equation). Due to the dilution of plasma volume by erythrocytes, the sensitivity to albumin concentration in full blood is lower than the sensitivity in plasma. The results of sensitivity analysis of phosphate concentration were negligible at this scale and are not shown.

**Fig. 3:**
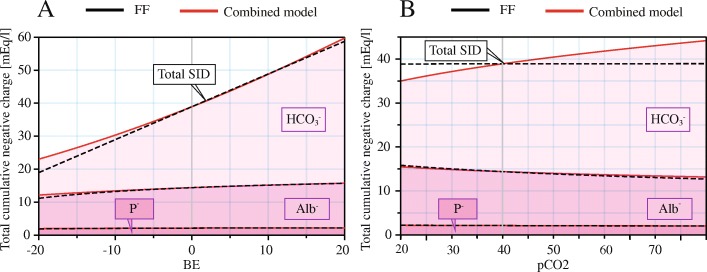
Composition of the SID. For various BE (**a**), i.e. addition of strong acid or bases, and during pCO_2_ titration (**b**) for the Figge-Fencl model of plasma (FF, black dashed) and our Combined model (red solid). The SID is no longer the independent variable in full blood

We can also use the Combined model to show the composition of the plasma SID. The differences of SID composition for pCO_2_ titration and for change in BE in the full blood and in the plasma is shown in Fig. 3. Note the different profiles for plasma and for full blood, especially during the change in pCO_2_ – the SID independence on pCO_2_ no longer holds in full blood (as confirmed in other literature, e.g. [21]). In Fig. 2 we use semi-logarithmic axis scale visualisation (pH vs log(pCO_2_)) to be consistent with the SA nomogram and the SID composition in mEq/l for variable pCO_2_ and BE (added or removed H^+^ ions) in Fig. 3 to compare with the Figge-Fencl model.

**Fig. 4:**
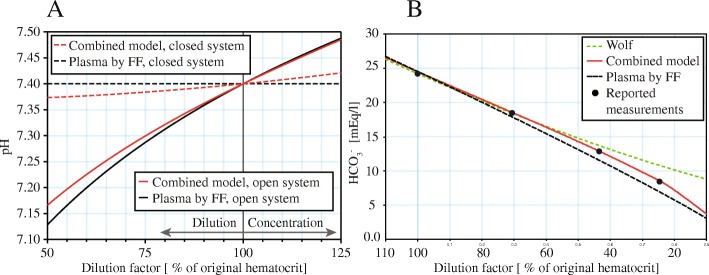
Prediction of dilution. **a** dilution with saline in the closed system, i.e. the pCO_2_ is also diluted (dashed) and in the open system, where the pCO_2_ is regulated to a constant level as in vivo (full line). **b** dilution predicted for the closed system – comparison with other models and with measured data, as reported in [21]

The model can predict the effect of dilution with saline solution. Figure 4a shows the comparison of the reaction of full blood versus sole plasma to dilution in the closed system (i.e. the total CO_2_ is also diluted) and the open system (constant pCO_2_, as in vivo) during dilution, represented as a percentage of the original hemoglobin content. Here, the pH of the closed system is no longer insensitive to the dilution and the the open system is additionally buffered by the erythrocytes. Plotting the dependency of the HCO_3_^−^ for the open system together with comparison to Figge-Fencl and recent Wolf [13] (reduced to plasma and erythrocyte compartments) models reveals, that the Combined model shows a perfect fit to measured values, as reproduced in [22] (Fig. 4b).

**Fig. 5:**
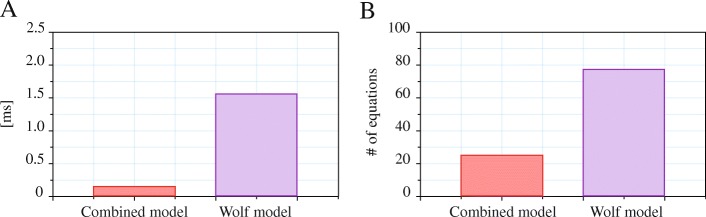
Computational complexity. CPU time needed for model initialization. The Combined model requires significantly lower CPU time for initialization (**a**) due to better numerical robustness than the latest version of the Wolf model (v3.51) reduced to P-E compartments and also has lower number of non-trivial equations (**b**)

The resulting Combined model is numerically stable within BE range of − 25 to 25 and, in the described combination, requires only 25 non-trivial (i.e. not direct value assignment) equations. In contrast, our implementation of the plasma-erythrocyte model by Wolf (version 3.51) has 77 non-trivial equations and its initialization problem consists of set of 64 non-linear equations (in contrast to only 13 of the Combined model). Our Combined model also needs significantly lower time for computation of the initialization problem (Fig. 5).

## Discussion

### Unification of modern and traditional approaches

The preferred bed-side approach to acid-base evaluation still remains under debate, for further information on this issue see [23–25]. A number of authors have strived to compare the traditional approach to the physicochemical, to be used at the bedside (e.g. [26, 27]) or even make use of both at once [28]. However, although different mathematical formalism, these two major approaches give similar information [29] and some studies conclude that, in principle, neither of the two methods offer a notable advantage [29–31].

Some previous works already attempted to find a relationship, e.g. Schlichtig [15] addressed the question of how base excess (BE) could have been increased, even though the strong ion difference (SID) had remained unchanged among hypoproteinaemia patients, by proposing SIDEx based on the SA approach to the Van Slyke eq. [9]. Wooten later demonstrated mathematically, that “BE and the change in SID are numerically the same for plasma, provided that the concentrations of plasma noncarbonate buffers remain constant” [16], Matousek continued in the mathematical comparison [29, 32]. We extend the comparison, which was made mostly for plasma, to full blood and variable albumin and phosphate levels and present a method of combining both traditional and physicochemical approaches. NSID also indicates the desired value of SID during albumin and phosphate disorders.

### The combined model

The resulting Combined model fits the full-blood Siggaard-Andersen model (by definition) and also the contemporary physicochemical model by Wolf [13] reduced to the plasma-erythrocyte compartment. Our contribution lies in the extending the classical physicochemical Stewart approach with the buffering effect of erythrocytes and their effects on SID during variable pCO_2_, while employing also the BE metric for quantization of H^+^/HCO_3_^−^ flow balances. The resulting model is computationally effective and the solution does not exhibit numerical problems within normal input ranges.

During the pCO_2_ titration (Fig. 3b), it can be seen that the SID of plasma in whole blood is not independent of pCO_2_ and therefore could not serve as an independent state property. On the contrary, the BE_OX_ (BE correction for virtual fully oxygenated blood, which could be calculated e.g. as in [33]), together with total O_2_ and total CO_2_ blood concentrations, are then a truly independent state properties of the full-blood component (as implemented in accompanied model [18]).

The concept of NSID, redefined for standard interstitium conditions, is then together with the eq. (5) usable also for calculations of BE in interstitial fluid to interconnect the blood with the interstitial compartment.

The proposed model does not depend on any new parameter or set any limitation to the contemporary approaches; rather, it solely joins them using the additional assumption of passive ion exchange only.

### Combined model assumptions and limitations

The Combined model relies on fundamental assumptions of each of the combined approaches. The erythrocytes in the HCT1 compartment provide an additional buffer, mostly due to the binding of H^+^ to hemoglobin. This is associated with interchange of Cl^−^ for HCO^−^ _3_, also during the change in pCO_2_, which has been described in numerous textbooks. The H^+^ / HCO_3_^−^ flows are not directly conserved, as it might be buffered by a number of mechanisms, but persists in the form of BE metric. In Stewart’s terms, the H^+^/HCO_3_^−^ flows are expressed in the form of SID change, i.e. here as exchange for Cl^−^.

To maintain the electroneutrality, we assume 1:1 transfer. As a complement of the reduced Cl^−^ in the plasma, the HCO_3_^−^ in Fig. 3b is rising and so is the total SID. Again, this could be viewed from two standpoints: in Siggaard-Andersen’s traditional approach it is the change of BE, e.g. + 1 M of HCO_3_^−^ equals the change of BE by plus one. In Stewart’s terms, this equals to the change in SID by + 1 (as shown by [5]). In the current case of HCO_3_^−^ to Cl^−^ exchange, it is decrease of Cl^−^ by exactly 1 M.

Our approach is generally limited by the measured data for behavioral model - the plasma is mechanistically extendable and replaceable by more complex models (see the next section), but the reactions in erythrocytes are measured for standard conditions only - that is normal concentration of DPG (5,0 mmol/l), fetal hemoglobin (0 mmol/l) and CO (0 mmol/l). To take these into account, the behavioral description would need to be radically extended by a number of dimensions, impairing the computational effectivity.

**Fig. 6:**
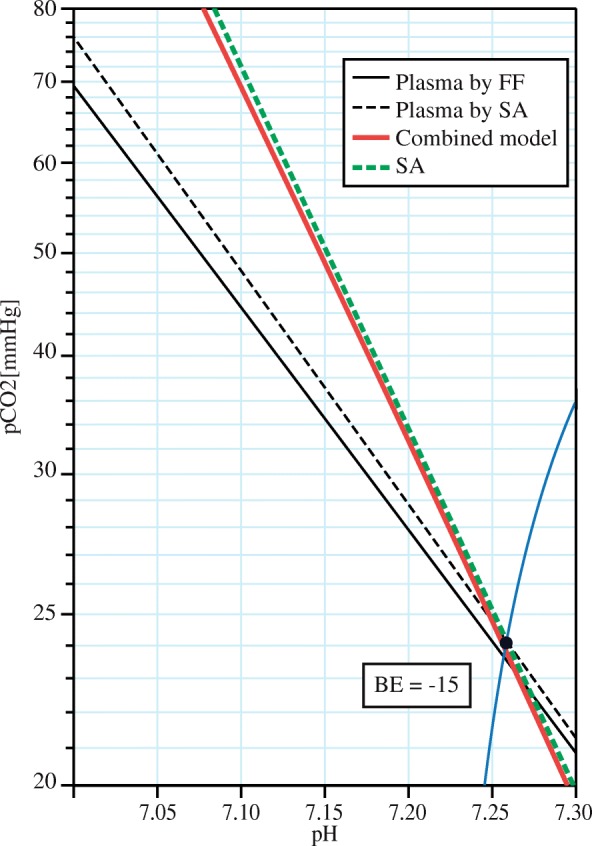
Magnification of Fig. 2a at BE = − 15: Disparity between the base models. The difference between Figge-Fencl (FF, black) and Siggaard-Andersen’s (SA, black dashed) models of plasma (blood with HCT0) is causing the Combined model (red bold) not being exactly aligned with the SA model (green bold dashed) even for otherwise standard conditions (hematocrit 0.45, normal levels of Alb and Pi)

The slight inaccuracy of the fit to Siggaard-Andersen’s model for very low BE values is caused by the inconsistency between the SA and the Figge-Fencl (FF) models (Fig. 6). The SA model, extrapolated to zero hematocrit, i.e. plasma, does not exactly fit the FF model in limit BE ranges. This error could possibly be caused by using the SA model outside the measured boundaries, while extrapolating hematocrit to zero or close to one (an unphysical state), although the linearity is one of the assumptions of SA. Yet, based on the literature data, we are unable to distinguish whether the error is caused by the extrapolation error, measurement inaccuracy of SA, or incompleteness of the FF model. Otherwise, for standard conditions (i.e. normal hematocrit, albumin, phosphates, and dilution) the Combined model performs according to Siggaard-Andersen.

### The various-complexity implementation

Our implementation in Modelica language enables switching complexity of a particular compartment. That is, we can use three different models for formulation of the physicochemical plasma compartment with various complexity: the simplest model of Fencl [34], the Figge-Fencl model [14], updated to version 3.0 [20] to quantify albumin in detail, or Wolf’s plasma compartment from [35] in current version v3.51 to consider also effects of Mg^2+^ and Ca^2+^ binding on albumin). The trade-off is computational complexity. For description of the Siggaard-Andersen erythrocytes compartment, we include the original formulation of the Van-Slyke eq. [9], our exact formalization using the set of four sixth-order polynomials and the simplest model of Zander [36]. Optionally, the computation can be enhanced with dilution. This allows to choose a simple to medium-complex description, based on current modeling needs. The full-blood component is supposed to be used in multiple arterial and venous parts of a complex model, thus the low complexity and robustness are vital.

### Computation complexity

Because the acid-base computations are highly non-linear, iterative algorithms which converge to the solution are often used (as employed by e. g. Siggaard-Andersen’s OSA [37], Figge-Fencl calculations [20] or even by the newest Wolf model [13] in the form of a running constraint-unknown optimization problem). From the authors observation, the algorithm may diverge and fail to find any solution or there might be more possible solutions to the formulation of the problem, therefore these models are often sensitive to the initial guess of unknown variables and could have problems for states far from the physiological norm (usually being the initial guess). This makes acid-base modeling particularly challenging, as small changes to guesses of state variables may lead to potentially invalid solution.

The numerical solvability of the equation-based models depends especially on the non-linearity of the problem, particularly on the Jacobian matrix condition number of the equation system. This is, however, very specific to the chosen tool and usage of the certain model and, regrettably, no such formal metric is currently established (F. Casella, personal communication, November 2017). For model comparison, we use a number of non-trivial equations, which might be a good indicator for overall model complexity. To assess the solvability, we propose to use the time needed to consistently initialize the equation set, that is to find exactly one solution. For harder tasks, the solver employs several iterative methods one after another to overcome the convergence problems caused by non-linearities, which will affect the initialization time. Sometimes, the solution may not be found. From our experience, these two metrics does not necessarily correspond to each other.

Wolf model [35] is the most complete mechanistic description of whole body acidbase. The model is available as a VisSim simulator, which is however unsuitable for integration into other models. Convergence to a solution of its original implementation takes approximately 5 - 20s on the reference computer. The computation time of our reimplementation in modern equation-based Modelica language is negligible (almost instant - see Fig. 5), however it uncovers numerical instabilities and thus this model, as a whole, is unsuitable to be a part of larger integrative model. Our Modelica implementation of Wolf’s E-P (erythrocyte - plasma) has 77 non-trivial (other than direct assignment) equations and is numerically hard to solve - some input settings (e.g. low pCO2 and high BE) lead to invalid solutions and some Modelica tools are even unable to compute the erythrocyte-plasma model correctly at all (yet does not have any problems with the proposed Combined model). Although we admire the level of detail of the Wolf’s mechanistic model, for our needs it is unnecessarily complex, even when reduced to E-P compartments.

### Albumin

The Combined model has been originally designed with focus on precise albumin computation (when the Figge-Fencl plasma model is employed) in combination with hemoglobine buffering. Although the albumin concentration is considered important, Figs. 2b and 3 suggest that the plasma protein buffer capacity is significantly lower than the buffer capacity of hemoglobin.

**Fig. 7:**
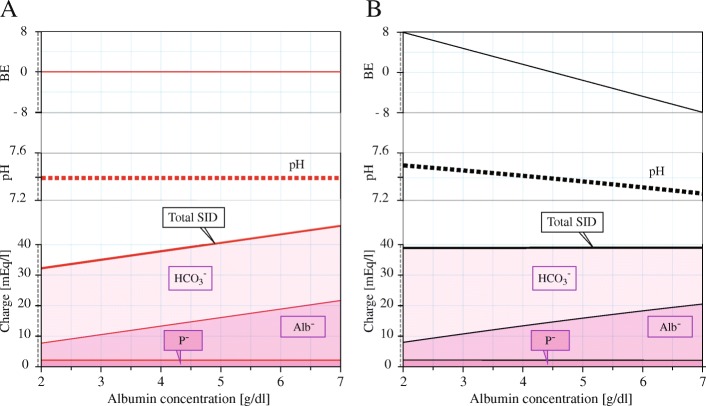
The effect of changing the albumin level in plasma. **a** Adding (or removing) the albumin together with its anti-charged cation equivalent (e.g., Na^+^) varies the the SID accordingly (bold red) and the BE and pH remain constant, which contrasts with clinical observations. **b** Adding (or removing) the albumin in electroneutral solution with corresponding amount of H^+^ maintains the SID unchanged (bold black), but the pH rises to alkalosis for hypoalbuminemia and falls to acidosis during hyperalbuminemia, which corresponds with Stewart’s theory of acute hypoalbuminemic alkalosis

However, the reaction to the albumin depletion may be different in vivo than what is presented during constant BE in Fig. 2b. In acute hypoalbuminemia, we can consider two cases of albumin depletion or addition, based on the electroneutrality assumption. The first is that an albumin is added or removed together with a cation (Na^+^, K^+^, etc.) in a neutral pH solution. This would change the SID (by the albumin charge), but keep the BE (and thus, the pH) constant (Fig. 7a). The second case is that albumin is bound with H+ only, in an acidic solution. As virtually each H^+^ recombines to form HCO_3_^−^, the loss of the albumin charge is compensated by the HCO_3_^−^ charge. Therefore, the SID is constant, but the BE (and thus, the pH) changes (Fig. 7b). The prediction of the BE change is made possible by the eq. (5).

Clinical observations favor the second case, where the SID is reported normal during hypoproteinemic alkalosis [38]. Some later studies [27, 39] challenge the existence of hypoproteinemic alkalosis in their study dataset, but we theorize that the effect (elevated BE) of albumin depletion in these studies has already been compensated. The exact explanation of this phenomenon is yet to be addressed by the full-body model, which would extend the currently presented model.

## Conclusion

When the inner details of any component are not the objective in complex integrative modeling, we can significantly reduce the complexity by substituting it with the behavioral description, yet still retain mechanistic properties of other components and its interactions.

We present a method to quantify the interconnection of two generally used and well-known approaches to acid-base balance, using no additional parameters or assumptions other than passive ion exchange.

The resulting Combined model of full-blood acid-base balance unites the advantages of each approach: it can simulate variations in albumin level, buffer the effect of erythrocytes and predict a reaction to hemodilution and hemoconcentration, yet remains computationally simple. On the other hand, the proposed approach is insensitive to non-normal DPG, HbF and CO concentrations.

The combination gives an additional insight to the acid-base balance by establishing the relationship between the SID and the BE (using the defined NSID in the eq. (5)).

The model is designed to have a variable computational complexity and to be effectively extended by other bodily compartments (interstitial fluid, intracellular fluid, metabolism) and regulations (respiratory and renal) to assess the whole-body dynamic acid-base status.

### Additional file

Additional file 1: Formalization of Siggaard-Andersen nomogram and derivation of the base excess for the physicochemical domain. (PDF 806 kb)

## Appendix

Listing of the simplest flat implementation of Combined model in Modelica. To keep the implementation lucid, the dilution is not incorporated here for the sake of simplicity of this example. In this example, the Fencl’s [14] approximation is used for plasma, whereas for erythrocytes here we use a simple formalization of Siggaard-Andersen nomogram by Zander and Lang [36]. Use any Modelica tool (e.g., OpenModelica) to run this model. For the complete set of used models, including sources for presented figures, implementation of the Wolf model and the complete full-blood component, please refer to [18].
