# Supplementary material for: Modern and traditional approaches combined into an effective gray-box mathematical model of full-blood acid-base
Source: Theor Biol Med Model. 2018 Sep 10;15:14. doi: 10.1186/s12976-018-0086-9 (PMC6130067; doi:10.1186/s12976-018-0086-9)
Supplement: Supplementary file 1 — Formalization of Siggaard-Andersen nomogram and derivation of the base excess for the physicochemical domain. (PDF 806 kb) [file 12976_2018_86_MOESM1_ESM.pdf]

Formalization of Siggaard-Andersen nomogram and derivation of the base excess for the physicochemical domain

A supplementary material to *Modern and traditional approaches combined into an effective gray-box mathematical model of full-blood acid-base* by Filip Ježek and Jiří Kofránek, 2018

Formalization of the Siggaard-Andersen nomogram

The Siggaard-Andersen nomogram [1] used to be solved graphically with a pH-pCO2 semi-logarithmic graph by finding appropriate values on the base excess (BE) and buffer base (BB) curves and connecting the two points by a line on which we read the pCO2 and pH values relationship. With the emergence of computers, the author of this nomogram then approximated it by the well-known Van Slyke equation ([2], further updated in [3]). To minimize both the approximation and extrapolation errors (especially for haemoglobin), we employ our own methodology to give an exact formalization of the original Siggaard-Andersen nomogram. Our approach is based on the graphical method of reading from the semi-logarithmic nomogram.

Because the haemoglobin (Hb) buffer strength depends also on oxygen saturation (sO2), we assume full oxygen saturation (sO2 = 1). Otherwise, the BE could be corrected to BE_{OX} (base excess of a full oxygenated blood), as described in [4]:

(1) $$BE_{ox} = BE - 0.2 \cdot (1 - sO2) \cdot Hb;$$ [mEq/l; mEq/l; -; g/100ml]

For further reference, we assume full saturation and thus BE_{OX} = BE. Then, the buffer base (BB) can be computed from BE as follows [1]:

(2) $$BB = BE + 0.42 \cdot Hb + 41.7;$$ [mEq/l; mEq/l; g /100ml]

These values are then used as an input to the BE and BB curves to provide pCO_2 and pH values. We formulate the functions of BB and BE curves and fit them to the original data of [5], as reproduced by [6], using one fourth and three sixth-order polynomials with the Matlab Curve Fitting Toolbox:

(3) $$f_{pCO_2BB}\left(BB\right) = \sum_{i=0}^{6} C_{pCO2BB_i} \cdot BB^i$$

(4) $$f_{pHBB}\left(BB\right) = \sum_{i=0}^{6} C_{pHBB_i} \cdot BB^i$$

(5) $$f_{pCO2BE}(BE) = \sum_{i=0}^{6} C_{pCO2BE_i} \cdot BE^i$$

(6) $$f_{pHBE}(BE) = \sum_{i=0}^{4} C_{pHBE_i} \cdot BE^i$$

	i=0	i=1	i=2	i=3	i=4	i=5	i=6
C_{pCO2BB_i}	-97.0551	8.2352	$-38.1\text{x}10^{-3}$	$-3.2\text{x}10^{-3}$	$72.7\text{x}10^{-6}$	$-640.9\text{x}10^{-9}$	$2.1\text{x}10^{-9}$
C_{pHBB_i}	39.9871	0.3537	$-46.5\text{x}10^{-3}$	$231.6\text{x}10^{-6}$	$3.8\text{x}10^{-6}$	$-513.9\text{x}10^{-9}$	$8.3\text{x}10^{-9}$
$C_{pCO_2BE_i}$	7.2888	-0.0678	$4.07\text{x}10^{-3}$	$-111.7\text{x}10^{-6}$	$1.6\text{x}10^{-6}$	$-13.0\text{x}10^{-9}$	$0.40\text{x}10^{-9}$
C_{pHBE_i}	7.4001	$11.9\text{x}10^{-3}$	$175.6\text{x}10^{-6}$	$2.5\text{x}10^{-6}$	$131.3\text{x}10^{-9}$		

Table 1: Parameter values for precise formalisation of the SA nomogram (eq. 3 - 6)

Given the equation of the line as

(7) $$y = mx + c$$

where *m* can be expressed from two measurement points (x1, y1) and (x2, y2) as:

(8) $$m = (y2 - y1)/(x2 - x1)$$

and the y-intercept c is given by the point (x1,y1) for x1 = 0, we derive

(9) $$y = \frac{(x - x1)\,(y2 - y1)}{(x2 - x1)} + y1$$

where the x values are logarithmic readings of pCO_2, whereas the y values are readings of pH. We then substitute the functions into the line equation, such that:

(10)

$$pH = \frac{(\log_{10} pCO_2 - \log_{10} f_{pCO2BB})(f_{pHBB} - f_{pHBE})}{(\log_{10} f_{pCO2BB} - \log_{10} f_{pCO2BE})} + f_{pHBB}$$

We can use any equation-based modeling language to derive the following function for pH from equations (2) - (10) and find a steady-state solution for the pH:

(11) $$pH_{SA} = f_{SA}(BE,\ pCO_2,\ Hb,\ sO_2)$$

This method of formalization of the SA nomogram provides a perfect fit with the original data, at the cost of more complex computations. Especially it does feature a correct linear asymptotic behavior for extreme values of Hb, which is critical for our approach.

For a graphical comparison to the single Van Slyke equation [2] and the equation provided by Zander [7], which is claimed to be even more precise [6], see Figure S1. The sum of the RMS error of our approach with the original data [8] is below 10^{-3} *[unitless]*. Thus, this expression of Siggaard-Andersen technique yields superior results as exact as the nomogram itself and does not introduce any additional error. The source code for the Modelica implementation is accessible at [9] in *FullBloodAcidBase.FullBloodEmpirical.SAoriginal*.

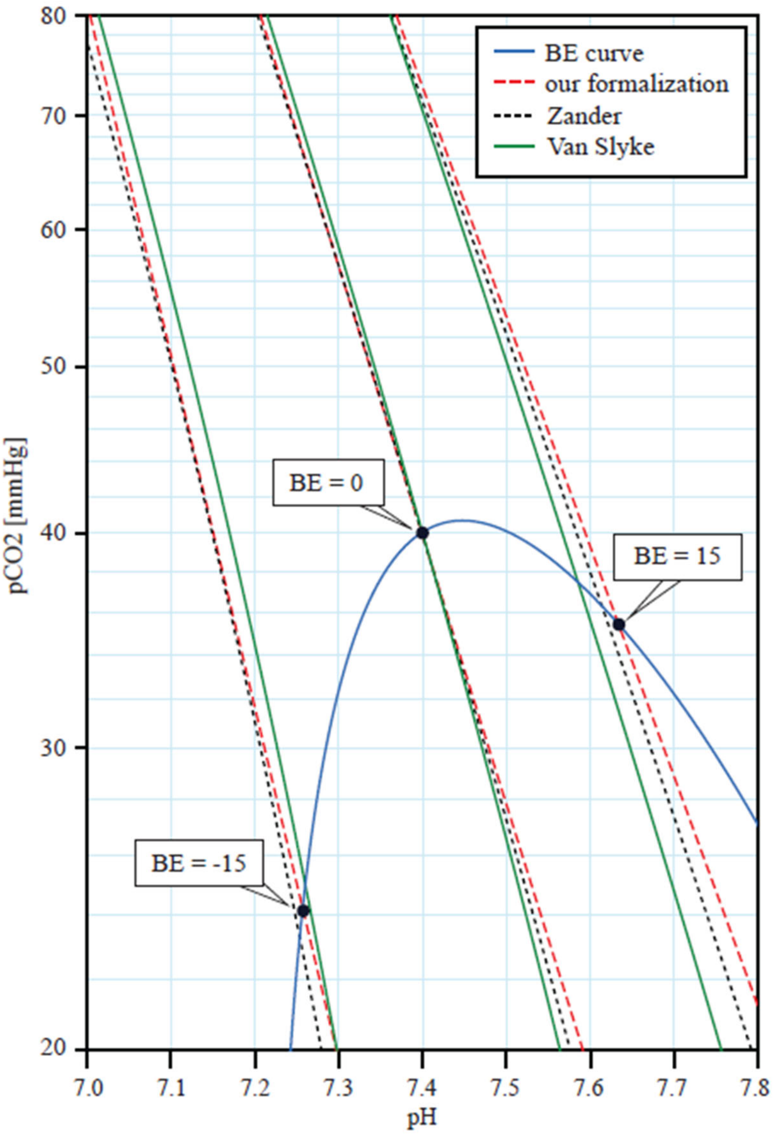

Figure S1: The result of our formalization in comparison to the "Van Slyke equation" formalization by Siggaard-Andersen [4] and to the formalization by Zander [7] for BE -15, 0 and 15.

Derivation of the Base Excess in the physico-chemical domain

We employ the model described by Fencl et al. [10]:

(12) $$HCO_3 = \frac{24.4 \times 10^{-9} \times pCO2}{10^{-pH}}$$

(13) $$P = -Pi \times (0.309\,pH - 0.469)$$

(14) $$\text{albuminCharge} = -(10Alb) \times (0.123pH - 0.631)$$

(15) $$SID + \text{albuminCharge} + P \ + (-1 \cdot HCO_3) = 0$$

albuminCharge, P and *SID* are the charge concentrations of the respective substances, expressed in [mEq/l], whereas *Alb* is the input albumin concentration in [g/dl] and *Pi* is the input phosphate concentration in [mmol/l]. To be consistent with the Fencl's formulation, HCO_3^- is also expressed as the concentration in [mmol/l]. Equations (11) – (14) form a functional expression for the calculation of pH from physico-chemical (further shortened to *PC*) model, which can be numerically solved by the Modelica tool to find a steady-state solution to the equations:

(16) $$pH_{PC} \ = \ f_{pH_{PC}}(SID,\ pCO_2,\ Pi,\ Alb)$$

For computation of SID, the Modelica language provides a straightforward reuse of the physicochemical model. We employ the same equations (11) - (14) and specify the pH as an input and SID as an output variable, such that:

(17) $$SID \ = \ f_{SID_{PC}}(pH,\ pCO_2,\ Pi,\ Alb)$$

For computation of NSID, we just specify the normal conditions as the inputs:

(18) $$NSID = f_{NSID_{PC}}(pH \ = \ 7.4,\ pCO2 = 40\ mmHg,\ Alb,\ Pi)$$

, where *Alb* [g/dl] and *Pi* [mmol/l] are the actual values. Then, the base excess for physicochemical model (BE_{PC}) reads:

(19) $$BE_{PC} \ = \ SID \ - \ NSID$$

For more precise yet computationally more demanding calculation, one might employ more detailed calculations of albumin and phosphate charge (equations 12 - 14), e.g. the Figge-Fencl model [11]. For implementation of both cases, as well as detailed physico-chemical approach by Wolf [12], see the model source code [9].

References

1. Andersen OS. The pH-log pCO2 blood acid-base nomogram revised. Scand J Clin Lab Invest. 1962;14:598–604.

2. Siggaard-Andersen O. The van Slyke equation. Scand J Clin Lab Invest Suppl. 1977;146:15–20.

3. Siggaard-Andersen O, Siggaard-Andersen M. The oxygen status algorithm: a computer program for calculating and displaying pH and blood gas data. Scand J Clin Lab Invest Suppl. 1990;203:29–45.

4. Siggaard-Andersen O. Textbook on the Acid-Base and Oxygen Status of the Blood. Acid-Base and Oxygen Status of the Blood. 2010. http://www.siggaard-andersen.dk/OsaTextbook.htm. Accessed 7 Jul 2016.

5. Siggaard-Andersen O, Others. The acid-base status of the blood. Munksgaard.; 1974.

6. Lang W, Zander R. The accuracy of calculated base excess in blood. Clin Chem Lab Med. 2002;40:404–10.

7. Zander R. Die korrekte Bestimmung des Base Excess (BE, mmol/l) im Blut. AINS-Anästhesiologie- Intensivmedizin- Notfallmedizin- Schmerztherapie. 1995;30 S 1:S36–8.

8. Siggaard-Andersen O, Others. The acid-base status of the blood. Munksgaard.; 1974.

9. Ježek F. full-blood-acidbase. Github. doi:10.5281/zenodo.1134853.

10. Fencl V, Jabor A, Kazda A, Figge J. Diagnosis of Metabolic Acid–Base Disturbances in Critically Ill Patients. Am J Respir Crit Care Med. 2000;162:2246–51.

11. Figge J. The Figge-Fencl Quantitative Physicochemical Model of Human Acid-Base Physiology (Version 3.0). Figge-Fencl.org - Figge-Fencl Quantitative Physicochemical Model of Human Acid-Base Physiology. 27 October, 2013. http://www.figge-fencl.org/model.html. Accessed 22 Jun 2016.

12. Wolf MB, DeLand EC. A comprehensive, computer-model-based approach for diagnosis and treatment of complex acid–base disorders in critically-ill patients. J Clin Monit Comput. 2011;25:353–64.
